# Lactylation in cancer: mechanistic insights, tumor microenvironment, and therapeutic horizons

**DOI:** 10.3389/fimmu.2025.1697008

**Published:** 2026-01-09

**Authors:** Qiang Yang, Zhibo Yang, Hai Zhao

**Affiliations:** 1 Department of Neurosurgery, The Second Affiliated Hospital of Lanzhou University, Lanzhou, Gansu, China; 2 Department of Neurosurgery, 3201 Hospital of Xi’an Jiaotong University Health Science Center, Hanzhong, Shaanxi, China; 3 Department of Neurosurgery, The Affiliated Hospital of Qingdao University, Qingdao, Shandong, China

**Keywords:** cancer immunotherapy, epigenetic regulation, histone modi7ication, immunometabolism, lactylation, therapeutic targets, tumor metabolism, tumor microenvironment

## Abstract

The discovery of lactylation, a post-translational modification derived from lactate, has fundamentally altered the perception of cancer metabolism. Once regarded as a metabolic waste product, lactate is now recognized as a central fuel source, a signaling molecule, and an epigenetic substrate capable of reprogramming gene expression and cellular function. Lactylation integrates metabolic reprogramming, tumor plasticity, and immune suppression, thereby orchestrating cancer initiation, progression, and resistance to therapy. This review provides a critical and integrative commentary on recent advances in lactylation biology, drawing from biochemical, epigenetic, and immunological perspectives. It synthesizes mechanistic insights into lactylation, highlights its role in tumorigenesis and the tumor microenvironment (TME), and evaluates therapeutic strategies that target lactate production, transport, and lactylation machinery. By dissecting consensus, controversies, and unresolved questions, we argue that lactylation represents both a hallmark of tumor adaptation and a potential Achilles’ heel for intervention. We further discuss future research directions, including comprehensive lactylome mapping, structural biology of lactylated proteins, microbiome-derived lactate, and clinical translation. Ultimately, lactylation is not merely a byproduct of glycolysis but a metabolic language that tumors employ to communicate, adapt, and thrive. Decoding this language may open new frontiers in cancer therapy.

## Introduction

**Figure 1:**
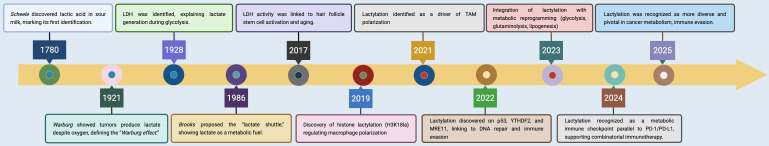
Milestones in lactate and lactylation research. This timeline summarizes key discoveries from the identification of lactic acid in 1780 to recent advances in 2025. Highlights include the Warburg effect, the lactate shuttle, and emerging roles of histone and non-histone lactylation in metabolism, immunity, and disease progression.

The *Warburg effect*, first described nearly a century ago, characterized the paradoxical preference of tumor cells for glycolysis even in the presence of oxygen (1, 2). For decades, this phenomenon reinforced the notion of lactate as a metabolic waste product, expelled from tumor cells into the extracellular environment to prevent acidosis and sustain glycolytic flux (3) (**Figure 1**). However, isotope tracing and metabolomic studies have since shattered this dogma. Lactate is now recognized as a major carbon source for oxidative phosphorylation and biosynthesis, frequently surpassing glucose in contribution to the tricarboxylic acid (TCA) cycle (4–6). This revelation repositions lactate as a central metabolite at the crossroads of energy production, redox regulation, and signaling.

The most transformative advance in this trajectory was the discovery of histone lysine lactylation (Kla) in 2019, which directly linked lactate metabolism to chromatin modification (7, 8). Lactylation demonstrated that lactate could function as an epigenetic substrate, modifying both histones and non-histone proteins to reprogram gene expression (9). Unlike acetylation or methylation, lactylation is acutely sensitive to metabolic flux, reflecting environmental conditions such as hypoxia, nutrient deprivation, and immune pressure (10). In cancer, where glycolysis is constitutively activated and lactate accumulates, lactylation emerges as a dynamic regulatory mechanism that confers adaptability, survival, and resistance (11).

Thus, lactylation represents more than a novel biochemical curiosity. It is a functional bridge between tumor metabolism and transcriptional control, providing mechanistic insight into how tumors integrate metabolic reprogramming with immune evasion and microenvironmental remodeling (12). The following sections critically examine these dimensions, moving from biochemical underpinnings to tumor initiation, TME remodeling, therapeutic strategies, controversies, and future perspectives.

## Biochemical and epigenetic foundations of lactylation

The biochemical foundation of lactylation is closely tied to cellular lactate metabolism, and multiple donor pathways have now been identified. In mammalian cells, lactoyl-CoA has been quantitatively detected using LC–MS–based metabolomics, confirming its role as a principal acyl donor for lysine L-lactylation. Moreover, a recent study identified GTPSCS as a nuclear lactoyl-CoA synthetase catalyzing its formation, linking metabolic flux to histone and non-histone protein modification (13–16). Lactyl-CoA derives from lactate, itself produced primarily through lactate dehydrogenase A (LDHA)-mediated reduction of pyruvate. Continuous glycolysis ensures abundant substrate, while transport through monocarboxylate transporters (MCT1 and MCT4) regulates intracellular and extracellular lactate pools (17). In hypoxic tumor cores, lactate exported by MCT4 is taken up by oxygenated tumor regions through MCT1, fueling oxidative phosphorylation in a process termed the “lactate shuttle” (18, 19). This metabolic symbiosis enhances tumor resilience and expands metabolic heterogeneity.

The enzymatic regulation of lactylation is becoming increasingly well defined as new biochemical evidence emerges. Early studies suggested that the acetyltransferase p300 may participate in catalyzing lysine lactylation, and subsequent *in vitro* enzymology has provided direct support for p300-mediated L-lactyl transfer to histone substrates (8). Although the lactyltransferase activity of CBP is less well characterized, accumulating results indicate that p300—and potentially CBP—can function as bona fide “writers” of lysine lactylation in addition to their classical acetyltransferase roles (20). These findings raise fundamental questions about acyl-substrate competition. Rather than a simple replacement of acetylation by lactylation, current evidence suggests that fluctuations in intracellular metabolic flux—particularly glycolysis-driven lactate accumulation—may alter the relative availability of acyl donors, thereby influencing the balance between acetyl-CoA– and lactyl-CoA–dependent lysine acylation. However, whether lactyl-CoA reaches concentrations sufficient to broadly rewrite acylation patterns *in vivo* remains an open question. Equally important, lactylation is now recognized as a reversible modification. Class I histone deacetylases (HDAC1–3) and several sirtuins (SIRT1–3) have been experimentally confirmed to remove lactyl groups from histone and non-histone substrates both *in vitro* and in cells, demonstrating bona fide delactylase activity (21). These multifunctional deacylases indicate that lactylation does not persist longer than other post-translational modifications by default; rather, its stability is determined by enzyme activity, metabolic context, and chromatin environment. At present, whether additional, dedicated substrate-specific delactylases exist remains a subject of active investigation.

Although histone lactylation and acetylation target the same lysine residues (e.g., H3K18, H4K12) and share writer enzymes, they exhibit fundamentally distinct temporal kinetics and functional roles. Acetylation typically functions as a stable epigenetic mark, maintaining an open chromatin architecture essential for housekeeping genes and steady-state proliferation (22). The intracellular pool of acetyl-CoA is tightly regulated and relatively constant under physiological conditions, ensuring that acetylation provides a sustained structural foundation for cellular identity and lineage maintenance.

In sharp contrast, lactylation is characterized by rapid temporal dynamics, acting as a real-time sensor of metabolic flux rather than a static memory. Unlike the steady supply of acetyl-CoA, the concentration of lactyl-CoA fluctuates acutely in response to environmental stressors such as hypoxia or glucose deprivation (16). Studies indicate that histone lactylation levels can spike within hours of glycolytic activation, directly coupling the cell’s instantaneous metabolic status to its transcriptional output (23). This ‘metabolic plasticity’ allows lactylation to function as an ‘immediate-early’ epigenetic mechanism. While acetylation secures the transcription of cell-cycle genes, lactylation preferentially initiates transient, adaptive programs—such as stress tolerance, wound healing, and immune evasion—that are downregulated once metabolic homeostasis is restored. Thus, the shift from acetylation to lactylation represents a switch from ‘growth mode’ to ‘survival mode’ in the metabolically stressed tumor microenvironment.

Recent multi-omics profiling revealed that histone lactylation (notably H3K18la, H4K12la) upregulates ARG1, VEGFA, and M2 macrophage–associated genes, thereby promoting immunosuppressive polarization in the tumor microenvironment (24, 25). In tumor cells, elevated lactylation enhances expression of glycolytic enzymes (e.g., LDHA, PKM2) and stemness-related genes (e.g., SOX2, NANOG), contributing to metabolic reprogramming and cancer progression (26). Conversely, acetylation often regulates cell-cycle and proliferation-related transcriptional programs, whereas lactylation links directly to metabolic and inflammatory gene networks, suggesting distinct but partially overlapping transcriptional landscapes. This evidence supports the notion that excess lactyl-CoA may compete with acetyl-CoA, thereby redirecting lysine modifications toward lactylation-dependent transcriptional programs that reinforce tumor aggressiveness and immune evasion.

The functional consequences of lactylation extend across epigenetic and non-epigenetic domains (14). Histone lactylation alters chromatin accessibility, enhances transcription of glycolytic enzymes, and promotes oncogenic pathways such as EMT and lineage plasticity (23, 27, 28). Non-histone lactylation modifies proteins such as p53, impairing tumor-suppressor function, or nucleolar proteins such as NCL, which regulate RNA splicing and MAPK signaling (10, 25, 29). This duality underscores lactylation as both a transcriptional reprogrammed and a post-translational tuner of protein stability and function. Lactylation of nucleolin has been shown to exert pro-tumorigenic effects through dual regulation of RNA metabolism and mitogenic signaling. Functionally, lactylated NCL enhances alternative RNA splicing of proto-oncogenic transcripts, promoting the inclusion of exons that stabilize mRNAs encoding proteins such as MYC, BCL2, and MCL1. This splicing pattern supports tumor cell proliferation and survival. In parallel, lactylated NCL directly interacts with MAPK cascade components, including RAF1 and ERK1/2, thereby stabilizing the MAPK signaling complex and prolonging its cytoplasmic retention. The sustained activation of MAPK signaling subsequently amplifies the transcription of downstream effectors (e.g., ELK1, c-Fos), further enhancing tumorigenic potential (30).

Although histone lactylation is considered a widespread acyl modification, accumulating evidence indicates that its deposition across the genome is spatially and transcriptionally selective (31). Genome-wide *ChIP-seq* analyses have revealed that lactylation marks, particularly H3K18la and H4K12la, are enriched at promoters of glycolytic enzyme genes such as LDHA, PKM2, PFK1, and ENO1 (32). Beyond the well-characterized H3K18la, recent high-resolution mapping has unveiled biologically distinct roles for site-specific lactylation. H3K9la has been identified as a critical mark at promoters of pluripotency genes (e.g., OCT4, SOX2), driving cellular reprogramming and stemness maintenance in varying tumor contexts (28, 33). In the gene body, H4K79la is increasingly recognized for its role in facilitating transcriptional elongation, distinguishing it from promoter-centric modifications. Furthermore, H4K91la appears to regulate chromatin stability and nucleosome dynamics, acting as a structural checkpoint during DNA replication and repair (34). These findings suggest that the ‘lactyl code’ is far more complex than initially appreciated, with distinct sites governing specific phases of the transcription cycle and chromatin architecture.

This selectivity appears to arise from the physical coupling of metabolic enzymes with chromatin-modifying complexes. LDHA and p300/CBP co-localize at glycolytic gene loci, where locally produced lactate fuels site-specific lactyl-CoA synthesis, enabling preferential histone lactylation (35). Moreover, transcription factors such as HIF-1α and c-Myc, which are strongly activated under hypoxia and oncogenic signaling, directly recruit p300/CBP to glycolytic promoters, further facilitating lactylation-dependent transcriptional activation. Together, these findings support a metabolite–chromatin microdomain model, in which localized lactate accumulation and enzyme–chromatin co-assembly confer functional specificity to an otherwise broad histone modification, thereby selectively amplifying glycolytic transcription programs that sustain the Warburg phenotype.

The lactylation of p53 during tumor initiation is not merely a passive consequence of increased lactate accumulation but reflects a selectively regulated enzymatic process (36). Under hypoxic or glycolytic stress, the accumulation of nuclear lactate and its conversion to lactyl-CoA provide sufficient substrate for histone acetyltransferase-like enzymes, primarily p300/CBP and GCN5, which have been shown to catalyze p53 lactylation at specific lysine residues (e.g., K370, K382) (37).

Moreover, oncogenic signaling (e.g., HIF-1α and PI3K–AKT–mTOR) enhances both p300 nuclear recruitment and its lactyltransferase activity, conferring site-specific modification of p53 rather than a global acylation effect (38). Functionally, this modification suppresses p53’s DNA-binding affinity and transcriptional activation of target genes (CDKN1A/p21, BAX), thereby dampening cell-cycle arrest and apoptosis during early oncogenic transformation. Thus, p53 lactylation represents a metabolic-epigenetic convergence point, where elevated lactate levels and enzyme reprogramming cooperate to attenuate tumor-suppressive signaling and facilitate tumor initiation.

**Figure 2:**
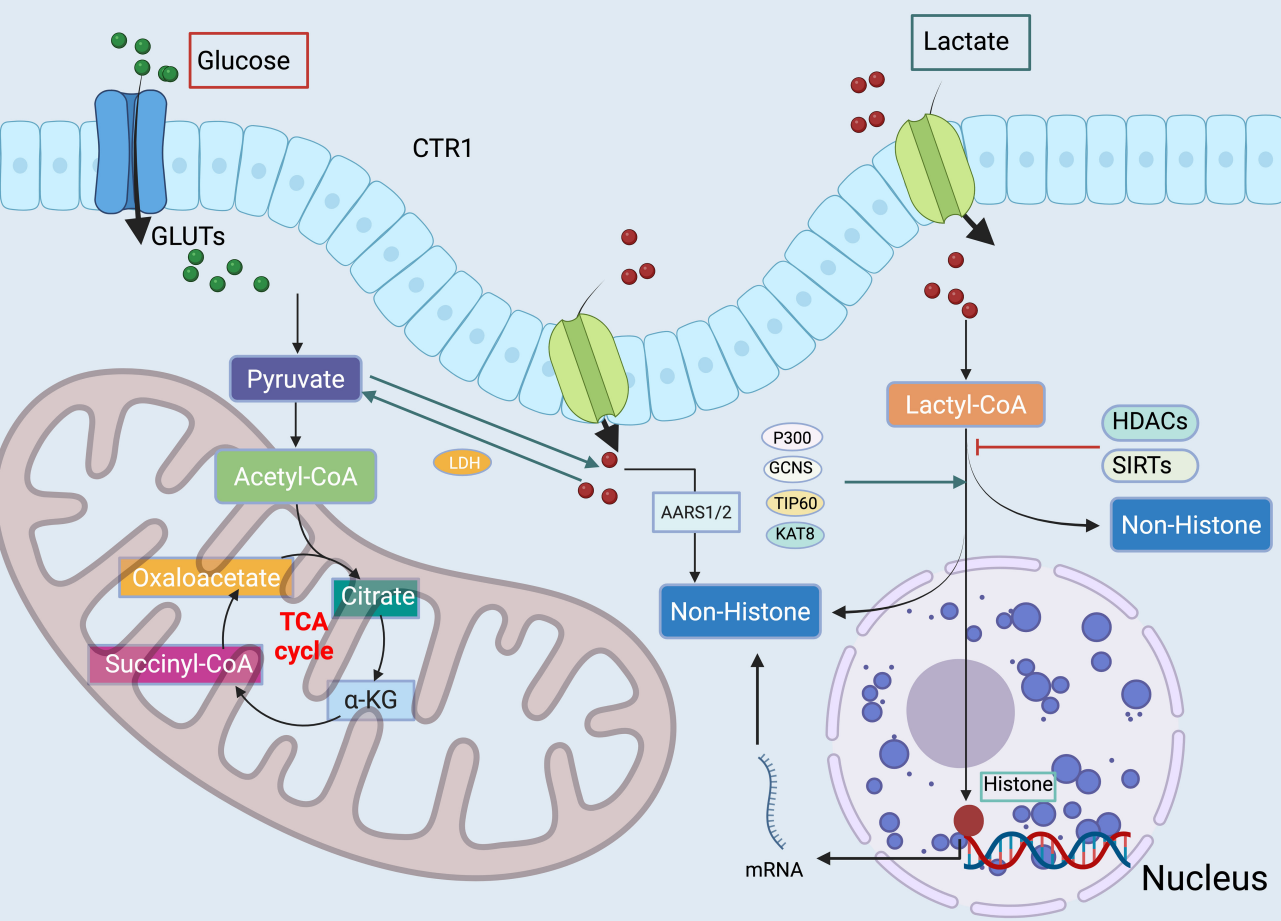
Interplay between lactate metabolism and protein lactylation. Lactate originates from both exogenous and endogenous sources. Exogenous lactate is transported into cells via monocarboxylate transporters (MCTs) and converted to lactyl-CoA, serving as a donor for histone and non-histone lactylation. Endogenous lactate arises from glycolysis, while under aerobic conditions pyruvate enters mitochondria to fuel the tricarboxylic acid (TCA) cycle.

Beyond p300/CBP, several additional enzymes have been implicated in catalyzing or facilitating protein lactylation. For instance, KAT8 (also known as MOF) and TIP60 (KAT5), two members of the MYST family of acetyltransferases, were reported to catalyze histone lactylation under hypoxic or high-glycolytic conditions (39–41). Moreover, aminoacyl-tRNA synthetases such as AARS1 and AARS2 have been suggested to participate in the non-canonical transfer of lactyl groups to lysine residues in cytosolic and mitochondrial proteins (40, 42). Although their catalytic efficiencies remain under investigation, these findings expand the repertoire of potential “writers” beyond the classical acyltransferases, supporting their inclusion in **Figure 2**.

## Lactylation in tumor initiation and progression

The initiation of cancer is characterized by the inactivation of tumor suppressors and the activation of oncogenes, processes in which lactylation is now implicated. Lactylation of p53 impairs its transcriptional activation of DNA damage response genes, weakening cell-cycle checkpoints and apoptosis (43, 44). This modification offers a mechanistic explanation for how p53 dysfunction may occur independently of genetic mutations, broadening the spectrum of p53 inactivation mechanisms in cancer. Similarly, lactylation of NCL alters alternative splicing and activates MAPK signaling, promoting proliferation and resistance (29).

Histone lactylation drives tumor plasticity and adaptability. In neuroendocrine prostate cancer, H3K18la increases LDHA expression and remodels chromatin near neuronal lineage genes, facilitating lineage plasticity and resistance to androgen deprivation therapy (45, 46). In other cancers, lactylation regulates EMT transcription factors such as ZEB1(Zinc finger E-box-binding homeobox 1), endowing tumor cells with migratory and invasive potential (47). This highlights lactylation as a dynamic enabler of phenotypic transitions that underlie metastasis and relapse.

Although both acetylation and lactylation can occur at H3K18, these two modifications are mutually exclusive at the same residue but can coexist in different nucleosomes or at distinct genomic loci in a context-dependent manner (48). In neuroendocrine prostate cancer, metabolic reprogramming driven by oncogenic signaling pathways such as MYC, PI3K–AKT–mTOR, and HIF-1α enhances aerobic glycolysis and lactate accumulation, leading to elevated levels of intracellular lactyl-CoA (49, 50). Under these conditions, the histone acetyltransferases p300/CBP—which can use lactyl-CoA as an alternative acyl donor—serve as major “writers” of H3K18 lactylation. This process represents not a passive replacement of acetylation but an active enzymatic shift that favors lactylation in regions of high lactate flux, particularly at promoters and enhancers of genes related to metabolism, lineage plasticity, and stress adaptation (51). In this metabolic and epigenetic context, H3K18ac and H3K18la display dynamic redistribution rather than a simple gain–loss pattern. H3K18ac remains associated with proliferation and housekeeping gene loci, while H3K18la is preferentially deposited at metabolic and stemness-related genes, creating partially overlapping yet functionally distinct transcriptional landscapes. This competitive occupancy allows lactylation to confer transcriptional selectivity and explains the selective activation of glycolytic and neuroendocrine programs observed in neuroendocrine prostate cancer (52).

The increase in H3K18la thus arises from both the enhanced nuclear availability of lactyl-CoA and the context-specific recruitment of p300/CBP to responsive chromatin regions. Potential delactylases such as HDAC1–3 and SIRT family enzymes may counterbalance this modification, although their precise roles in neuroendocrine prostate cancer remain to be elucidated (53). Regarding ZEB1 regulation, studies in epithelial–mesenchymal transition and cancer stemness models indicate that H3K18la and H3K9la enrichment at ZEB1 regulatory elements correlates with transcriptional activation (46). While the exact histone residue responsible for this regulation in neuroendocrine prostate cancer has not been unequivocally mapped, current evidence supports a model in which lactylation at H3K18 or H3K9 promotes ZEB1 expression and facilitates lineage plasticity during tumor progression.

**Figure 3:**
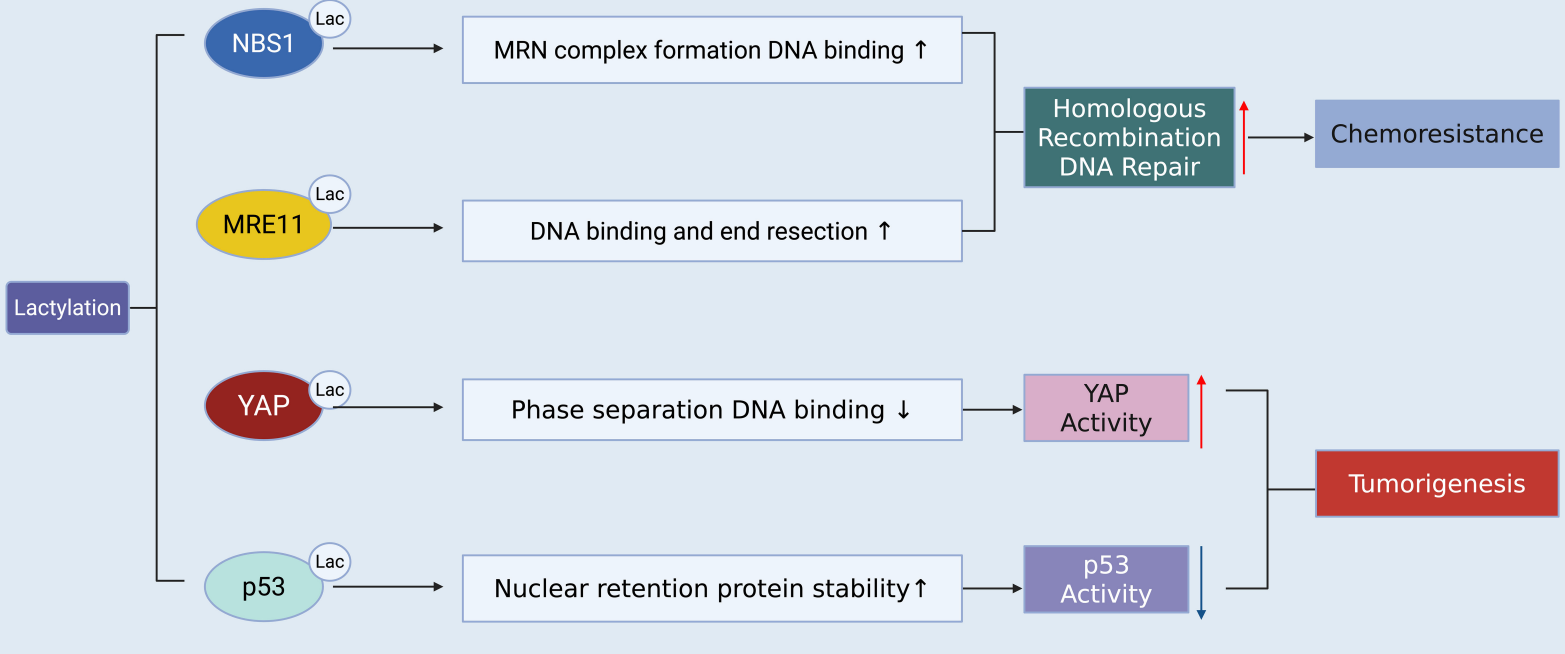
Mechanistic pathways linking lactylation to tumorigenesis and chemoresistance.

Equally important is the role of lactylation in therapy resistance. Lactylation-induced stabilization of HIF-1α promotes continued glycolysis and angiogenesis, sustaining tumors under therapeutic stress (32, 54). Lactylation also interfaces with drug efflux pumps and DNA repair proteins, further enhancing resistance (55, 56). Taken together, these findings underscore lactylation as a central driver of oncogenesis, plasticity, and resilience, with implications for early tumor initiation and late-stage progression ***(*****Figure 3*****)***.

The elevated levels of protein lactylation observed in cancer originate from the profound metabolic reprogramming that characterizes malignant cells (57, 58). The primary driver is the *Warburg effect*, in which persistent aerobic glycolysis leads to excessive lactate accumulation even under normoxic conditions (59). The elevated intracellular lactate pool serves as a substrate for lactyl-CoA synthesis, fueling histone and non-histone lactylation through p300/CBP and other acyltransferases (60). Beyond glycolytic flux, mitochondrial dysfunction and HIF-1α activation further amplify lactate production by enhancing LDHA expression and suppressing pyruvate oxidation. In parallel, monocarboxylate transporters (MCT1/4) dynamically redistribute lactate across tumor and stromal compartments, ensuring a constant supply of this metabolite for lactylation (61).

Recent advances have considerably expanded the catalog of lactylation-regulated proteins implicated in tumorigenesis, revealing that this post-translational modification influences a diverse array of cellular pathways that underlie malignant transformation. A pivotal example is HIF-1α, a central transcriptional regulator of metabolic adaptation. Under hypoxic conditions, tumor cells accumulate lactate due to enhanced glycolysis, and HIF-1α lactylation stabilizes the protein by reducing its ubiquitination and proteasomal degradation. This stabilization amplifies glycolytic gene transcription, vascular endothelial growth factor (VEGF) expression, and angiogenic signaling, forming a self-reinforcing metabolic loop that sustains tumor growth in hypoxic niches (32).

Another key node is pyruvate kinase M2 (PKM2), an enzyme critical for the Warburg effect. Lactylation of PKM2 on lysine residues modulates its structural conformation and subcellular localization. While unmodified PKM2 primarily drives glycolytic flux, its lactylated form translocates into the nucleus, where it serves as a coactivator for oncogenic transcription factors such as c-Myc and STAT3. This nuclear function enhances the transcription of glycolytic enzymes and cell-cycle regulators, reinforcing metabolic reprogramming and proliferation. Importantly, inhibition of PKM2 lactylation in preclinical models has been shown to reverse the Warburg phenotype and attenuate tumor aggressiveness, highlighting it as a potential therapeutic target (62).

Tumor suppressor p53, in contrast, represents an example of how lactylation undermines anti-tumor defenses. Lactylation of p53 at specific lysine residues—particularly within its DNA-binding domain—impairs its ability to transactivate downstream genes involved in cell-cycle arrest and apoptosis, such as p21 and BAX. Mechanistically, this modification reduces p53’s chromatin-binding affinity, resulting in diminished tumor-suppressive transcriptional activity and promoting oncogenic survival signaling. The interplay between acetylation and lactylation of p53 suggests that elevated lactate levels may tilt the post-translational balance toward a pro-tumorigenic state (44).

Similarly, YTHDF2 has recently been identified as a lactylation-sensitive oncogenic regulator. Lactylation enhances YTHDF2 expression and stability, thereby accelerating the degradation of tumor-suppressive mRNAs that contain m6A modifications. This modification-driven turnover of key inhibitory transcripts promotes tumor cell proliferation and immune evasion. The lactate–YTHDF2 axis thus represents a critical convergence point between metabolic signaling and RNA epigenetics, offering new opportunities for therapeutic intervention in glycolytic tumors (63, 64).

Beyond transcriptional and epigenetic regulation, lactylation also governs genomic stability and therapy response. MRE11 and NBS1, essential components of the MRN complex responsible for double-strand DNA break repair, undergo lactylation that enhances their nuclease activity and recruitment to damage sites. This modification facilitates efficient DNA repair and confers resistance to genotoxic stress, including radiation and platinum-based chemotherapies. Consequently, tumor cells with hyperlactylated MRE11/NBS1 display marked chemoresistance, and pharmacologic blockade of lactate production (via LDHA inhibition) restores therapeutic sensitivity (65, 66). In parallel, high-mobility group box 1 (HMGB1)—a chromatin-associated protein and extracellular alarmin—has been shown to undergo lactylation, which augments its translocation and secretion. Lactylated HMGB1 promotes the expression of proinflammatory cytokines and the recruitment of immunosuppressive myeloid cells, thereby linking lactate metabolism to the inflammatory TME. This reinforces a positive feedback loop between lactate accumulation, immune suppression, and tumor progression (67, 68).

Collectively, these emerging findings demonstrate that lactylation is not a passive byproduct of altered metabolism but rather a dynamic signaling mechanism that orchestrates metabolic adaptation, transcriptional reprogramming, genomic maintenance, and immune evasion. Through the coordinated modification of proteins such as HIF-1α, PKM2, p53, YTHDF2, MRE11/NBS1, and HMGB1, lactylation integrates oncogenic metabolism with tumor-promoting transcriptional and epigenetic circuits. Understanding these multifaceted roles provides not only a unifying framework for how metabolic reprogramming drives malignancy but also a rationale for targeting lactylation-dependent pathways in precision oncology. Emerging evidence suggests that oncogenic signaling pathways such as MYC, PI3K–AKT–mTOR, and KRAS mutations promote glycolytic enzyme expression and increase the cytosolic NADH/NAD^+^ ratio, further stabilizing lactate accumulation (69, 70). Epigenetic feedback loops exacerbate this state: lactylation of histones at glycolytic gene promoters (e.g., LDHA, PFK1) enhances their own transcription, creating a self-reinforcing circuit of hyperlactylation (35). Finally, tumor–stroma metabolic crosstalk also contributes. Cancer-associated fibroblasts and immune cells within the tumor microenvironment secrete lactate that can be imported by cancer cells or myeloid cells, sustaining the lactylation landscape across the entire tumor ecosystem (71). Collectively, these processes constitute a metabolic–epigenetic axis, whereby dysregulated glycolysis and lactate flux directly drive aberrant protein lactylation and malignant adaptation.

## Lactylation and remodeling of the tumor microenvironment

The tumor microenvironment is increasingly recognized as a decisive determinant of cancer progression and therapy response (71–73). Lactylation plays a pivotal role in reprogramming immune and stromal components of the TME, thereby orchestrating an ecosystem that favors tumor survival (74). The elevated lactate in the TME originates primarily from aerobic glycolysis in rapidly proliferating tumor cells, a hallmark of the *Warburg effect*. Tumor cells convert glucose to lactate even under normoxic conditions, leading to a substantial accumulation of extracellular lactate. This lactate is exported through monocarboxylate transporters (MCT4 and MCT1) and acidifies the surrounding milieu, which profoundly shapes immune cell behavior (75). Once released, lactate is taken up by neighboring immune cells—such as macrophages, dendritic cells, and T cells—via MCT1, serving as both a metabolic substrate and a signaling molecule. Inside these immune cells, lactate can be converted into lactyl-CoA, which drives histone and non-histone lactylation to reprogram their functions toward immunosuppressive phenotypes (16).

In addition to tumor-derived lactate, certain immune subsets, including TAMs and myeloid-derived suppressor cells (MDSCs), can generate lactate intrinsically through glycolytic activation under hypoxia and inflammatory stimulation (76, 77). These immune-derived lactate pools may contribute to autocrine or paracrine lactylation, amplifying immunosuppressive feedback loops within the TME. Therefore, lactate accumulation in the TME represents a bidirectional metabolic exchange, dominated by tumor glycolysis but reinforced by immune cell metabolism, collectively sustaining a tolerogenic, pro-tumor environment.

Macrophages represent the most extensively studied immune population influenced by lactylation (78). High lactate concentrations drive histone lactylation in macrophages, skewing them toward an M2 immunosuppressive phenotype characterized by upregulation of anti-inflammatory cytokines and angiogenic factors (27). This epigenetic reprogramming sustains immune evasion and promotes vascularization. T cells and NK cells are likewise affected: lactate accumulation suppresses their cytotoxic activity, while lactylation of immune regulatory proteins may further consolidate their dysfunctional state (79, 80). Thus, lactylation acts as a metabolic immune checkpoint, functioning in parallel with PD-1/PD-L1 and CTLA-4 pathways (81, 82). While classical immune checkpoints such as PD-1/PD-L1 and CTLA-4 suppress antitumor immunity through receptor–ligand interactions that inhibit T-cell activation, lactylation functions as a “metabolic immune checkpoint” in a parallel but mechanistically distinct manner (83). Rather than blocking receptor signaling, lactylation remodels the metabolic and epigenetic landscape of both immune and tumor cells, establishing a suppressive tumor microenvironment. Specifically, lactate accumulation and subsequent histone and non-histone lactylation in macrophages promote polarization toward the M2-like immunosuppressive phenotype, characterized by high ARG1, IL-10, and VEGF expression (84). In T cells, elevated extracellular lactate impairs glycolysis and effector cytokine production, while histone lactylation in dendritic cells attenuates antigen presentation (85). Thus, lactylation and classical checkpoints converge functionally by restraining cytotoxic immunity yet act through distinct layers of regulation—one via metabolic–epigenetic reprogramming, the other via surface receptor signaling (14). The two axes may even cooperate: PD-L1 expression can be up-regulated under high-lactate conditions, suggesting that metabolic checkpoints such as lactylation can potentiate classical immune inhibitory pathways. This conceptual parallel underscores lactylation’s role as a fundamental regulator of immune tolerance in the tumor microenvironment.

**Figure 4:**
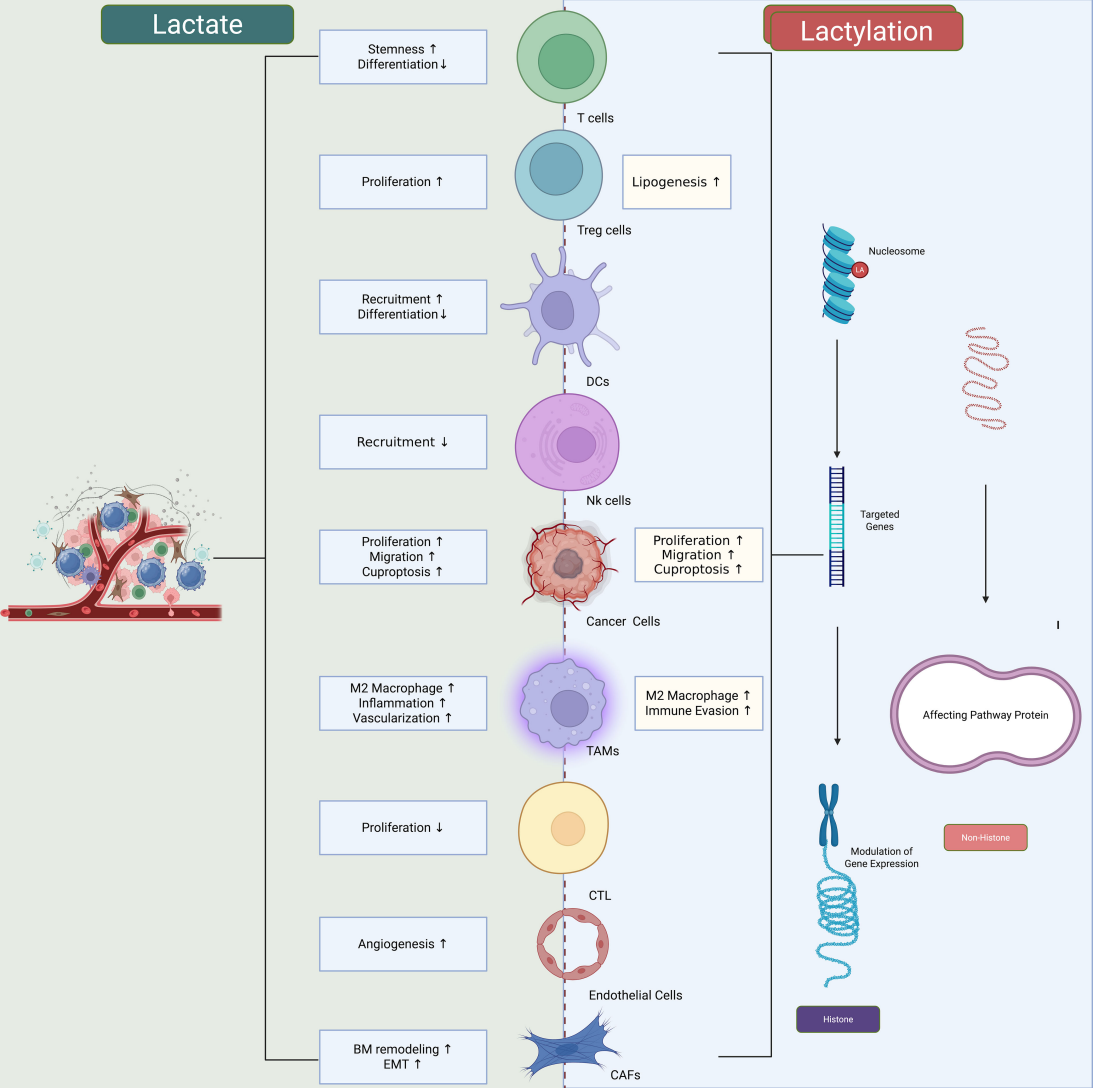
Lactate and lactylation as therapeutic vulnerabilities in cancer. Targeting lactate and lactylation represents a critical therapeutic vulnerability in cancer. Interventions directed against lactate/lactylation pathways can attenuate their role in oncogenic metabolic reprogramming and remodeling of the tumor microenvironment, highlighting their potential as an Achilles’ heel for effective cancer treatment.

Stromal fibroblasts and endothelial cells are also subject to lactylation-mediated reprogramming (86). In fibroblasts, lactylation enhances extracellular matrix remodeling, facilitating invasion and metastasis (86). In endothelial cells, lactylation promotes VEGFA expression and vascular permeability, supporting angiogenesis (32, 87). Together, these processes create a TME characterized by immune tolerance, stromal support, and enhanced vascular supply, all underpinned by lactylation as a unifying mechanism (**Figure 4**).

Lactylation enhances oncogenic activity by upregulating YAP signaling while impairing the tumor-suppressive function of p53. In parallel, modification of MRE11 and NBS1 within the MRN complex strengthens homologous recombination–mediated DNA repair, thereby driving chemoresistance.

## Systemic roles of lactylation across cancer biology

The biochemical foundation of lactylation lies in the cellular metabolism of lactate. As illustrated in **Figure 2**, both glycolytic and mitochondrial pathways converge on the formation of lactyl-CoA, which serves as the activated donor for lysine lactylation. Understanding this metabolic context is essential to appreciating how lactate availability regulates histone and non-histone protein modifications in cancer biology. While lactylation is often framed in the context of localized tumor metabolism, it is increasingly evident that its influence extends systemically, orchestrating metabolic adaptation across organs. Tumors act not as isolated metabolic islands but as nodes within a host-wide metabolic network. Lactate produced in tumors can circulate systemically, serving as a substrate for the liver, heart, and even the brain (88–90). This systemic lactate flux raises the possibility that lactylation might occur beyond the tumor boundaries, reshaping the biology of distant tissues.

Evidence from glioblastoma models indicates that lactylation contributes to the maintenance of glioma stem-like cells, endowing them with self-renewal and therapeutic resistance (91–94). In breast cancer, lactylation has been linked to estrogen receptor signaling, further connecting metabolic stress with hormone-driven proliferation (95–97). Lung cancers exhibit pronounced MCT1-mediated lactate utilization, with isotopic tracing studies demonstrating that lactate-derived TCA metabolites outcompete glucose-derived carbons (98, 99). In prostate cancer, lactylation supports the neuroendocrine phenotype and facilitates androgen independence, while in hepatocellular carcinoma it drives angiogenesis and immune suppression (100, 101). These tumor-specific manifestations underscore the context-dependent versatility of lactylation.

Beyond tumor-specific roles, lactylation intersects with lipid and amino acid metabolism. Lactate promotes glutaminolysis through c-Myc transcriptional regulation, enhancing the supply of α-ketoglutarate to the TCA cycle (23, 102). It also supports lipid biosynthesis by fueling acetyl-CoA pools and activating fatty acid synthase. Through these pathways, lactylation integrates diverse metabolic streams into oncogenic programs. Importantly, the convergence of lactylation with amino acid and lipid metabolism suggests that lactylation functions as a metabolic hub, directing nutrient utilization to optimize tumor fitness under stress (**Figure 2**).

Mechanistically, lactate regulates these anabolic processes through dual and complementary mechanisms. First, lactate acts as a signaling metabolite, stabilizing HIF-1α and activating c-Myc transcriptional programs that increase GLS1 and FASN expression, thereby sustaining glutaminolysis and lipogenesis (103). Lactate can also signal through G-protein-coupled receptor 81 (GPR81), which engages PI3K–AKT–mTOR signaling to further enhance lipid synthesis (104). Second, lactate serves as a substrate for histone and non-histone lactylation, providing an epigenetic layer of metabolic control. H3K18la and H4K12la enrichment at metabolic gene promoters opens chromatin and facilitates the recruitment of c-Myc, PPARγ(Peroxisome Proliferator-Activated Receptor Gamma), and SREBP1(Sterol Regulatory Element-Binding Protein 1), amplifying transcription of lipid- and amino-acid-metabolizing enzymes (105). Non-histone lactylation of enzymes such as PKM2 (Pyruvate Kinase M2 Isoform), ENO1 (Alpha-Enolase 1), and ACLY (ATP Citrate Lyase) has also been reported to boost glycolytic and lipogenic flux (106). Together, these findings indicate that lactate coordinates metabolic reprogramming through both signaling-dependent and lactylation-mediated mechanisms.

## Therapeutic targeting of lactate and lactylation

Therapeutic efforts to exploit lactylation biology fall into three categories: inhibition of lactate production, blockade of lactate transport, and modulation of lactylation-specific enzymes. Each strategy presents distinct opportunities and limitations.

Targeting lactate production has long focused on LDHA, the primary enzyme catalyzing pyruvate reduction to lactate (107). LDHA inhibitors reduce lactate accumulation but face challenges of systemic toxicity and compensatory metabolic pathways (108). Nevertheless, LDHA remains an attractive target, particularly in tumors heavily dependent on glycolysis. Targeting LDHB, which catalyzes lactate oxidation, has also been proposed in cancers reliant on lactate as fuel (109).

Lactate transport represents a more advanced translational strategy. MCT1 and MCT4 are key transporters that maintain lactate homeostasis in tumors (110, 111). AZD3965, a selective MCT1 inhibitor, has reached phase I/II clinical trials for advanced solid tumors and non-Hodgkin lymphoma (112). Preliminary results show tolerability and pharmacodynamic evidence of target engagement, though efficacy outcomes remain under evaluation. The development of MCT4 inhibitors, such as VB124, aims to disrupt lactate efflux from hypoxic tumor cells, collapsing the metabolic symbiosis that sustains tumor heterogeneity (113) (**Table 1**).

**Table 1:** Representative clinical trials targeting lactate metabolism and related pathways in cancer.

Target	Intervention	Mechanism of action	Indication	Phase	Trial identifier
MCT1	AZD3965	Inhibits lactate transport (import/export)	Advanced Solid Tumors, DLBCL	Phase I	NCT01791595
LDHA	NC-3200	Inhibits conversion of pyruvate to lactate	Advanced Solid Tumors	Pre-clinical/Early Phase	N/A
MCT4	VB124	Inhibits lactate efflux in hypoxic cells	Metastatic Pancreatic Cancer	Phase I	NCT06400000
p300/CBP	CCS1477	Bromodomain inhibitor (modulates lactylation writing)	Prostate Cancer, Hematologic Malignancies	Phase I/IIa	NCT03568656
Metabolism	Metformin + Chemo	Modulates systemic metabolism/OXPHOS	Breast Cancer, Lung Cancer	Phase III	NCT01101438

MCT1, Monocarboxylate Transporter 1; MCT4, Monocarboxylate Transporter 4; LDHA, Lactate Dehydrogenase A; DLBCL, Diffuse Large B-Cell Lymphoma; CBP, CREB-binding protein; OXPHOS, Oxidative Phosphorylation.

The third approach involves direct modulation of lactylation enzymes. p300/CBP inhibitors such as SGC-CBP30 and PLX51107 reduce histone lactylation and attenuate tumor growth in preclinical models (114, 115). HDAC and sirtuin inhibitors, already deployed in cancer therapy, may function as “de-lactylases,” though their lack of specificity complicates interpretation (115, 116). The identification of bona fide lactylation-specific erasers would represent a transformative advance, enabling highly targeted interventions.

Perhaps the most compelling therapeutic avenue is combinatorial. Lactylation-induced immune suppression creates a rationale for pairing lactylation-targeted therapies with immune checkpoint inhibitors (ICIs) (79). Preclinical studies suggest that blocking lactate production or transport reactivates T cell function, sensitizing tumors to PD-1/PD-L1 blockade. Similarly, lactylation-targeted therapies may enhance CAR-T and NK cell therapies by alleviating metabolic constraints in the TME (79). Thus, lactylation targeting is unlikely to succeed as monotherapy but may play a decisive role in multi-pronged immunometabolism strategies.

Recent preclinical evidence suggests that the relationship between PD-1/PD-L1 signaling, and lactate metabolism is bidirectional (117, 118). While blocking lactate production or transport reactivates T-cell function and enhances responsiveness to immune checkpoint inhibitors, PD-1/PD-L1 blockade itself can also influence lactate metabolism (119). Several studies have demonstrated that anti–PD-1 or anti–PD-L1 therapy partially restores glycolytic activity in effector T cells, increasing glucose uptake and lactate release within the tumor microenvironment (118–120). However, this T-cell–derived lactate surge does not mirror the immunosuppressive lactate generated by tumor cells; instead, it reflects a metabolic rejuvenation that supports T-cell proliferation and cytokine production.

Conversely, tumor cells under PD-1/PD-L1 blockade often experience metabolic stress due to reactivated immunity and interferon signaling (121). This stress can transiently upregulate LDHA expression and activity, potentially increasing lactate output in resistant tumors as an adaptive survival mechanism (120). Such findings indicate that blocking PD-1/PD-L1 may dynamically reshape lactate metabolism on both immune and tumor sides, emphasizing the metabolic plasticity of the tumor microenvironment. Consequently, co-targeting lactylation and PD-1/PD-L1 pathways may overcome adaptive resistance by simultaneously disrupting tumor glycolysis and restoring immune effector function, providing a strong mechanistic rationale for combinatorial immunometabolic therapy.

**Figure 5:**
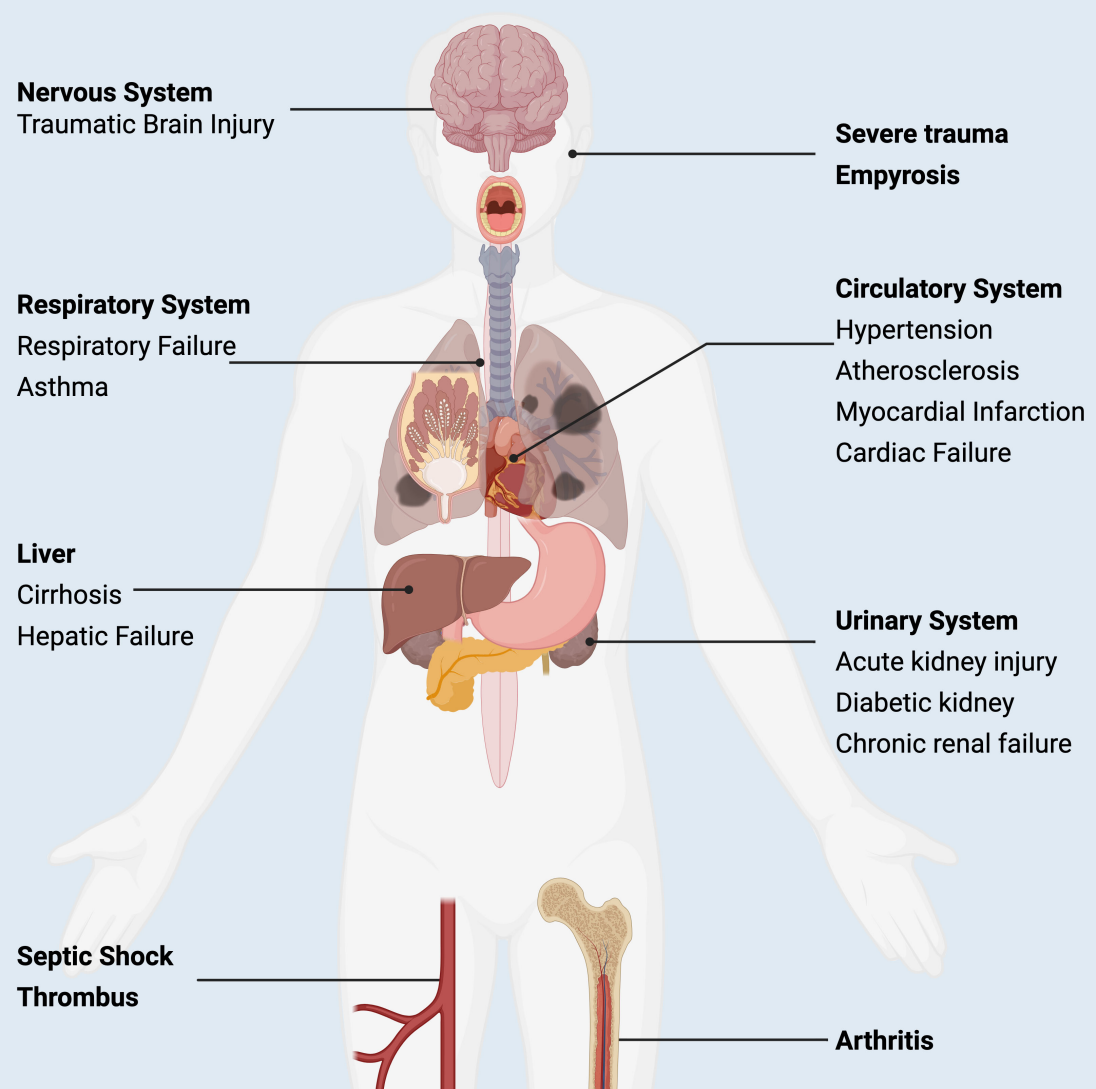
Pathophysiological roles of lactate across human diseases. Lactate has been implicated in the pathogenesis of a wide range of disorders, influencing the cardiovascular, respiratory, hepatic, urinary, and other organ systems. Beyond its metabolic functions, lactate serves as an important biomarker in the clinical diagnosis, monitoring, and prognosis of diverse diseases.

Beyond cancer metabolism, lactate accumulation has broad systemic effects, as shown in **Figure 5**. Excess lactate contributes to pathological remodeling in multiple organ systems—such as the cardiovascular, respiratory, hepatic, and nervous systems—highlighting the translational relevance of lactate biology across diverse disease contexts. This systemic perspective strengthens the rationale for targeting lactylation therapeutically in cancer (**Figure 5**).

## Consensus, controversies, and knowledge gaps

Several points of consensus have emerged across the literature. Lactylation is now firmly recognized as a critical link between metabolism and epigenetics (10, 122). Lactate functions not only as a fuel but also as a signaling molecule that drives epigenetic reprogramming and immune suppression (93, 123). Targeting lactate metabolism and lactylation holds promise for therapeutic intervention, with early-phase clinical trials underway.

Yet substantial controversies remain. One major debate concerns whether lactylation uniformly promotes tumor progression or whether it may exert context-specific suppressive roles. For example, some studies suggest that lactylation can stabilize pro-inflammatory gene expression in macrophages, potentially enhancing immune surveillance under certain conditions (27, 124, 125). This raises the possibility that lactylation may play dual roles, promoting or restraining cancer depending on cellular context and environmental cues.

Another unresolved issue is the existence of dedicated de-lactylases. Current evidence implicates HDACs and sirtuins in lactyl group removal, but whether these enzymes act specifically or incidentally remains unclear (126). The discovery of specialized lactylation erasers would reshape therapeutic strategies and provide tools for dissecting lactylation biology.

The temporal sequence of lactylation within the TME also remains contested. Does lactylation first occur in tumor cells, reshaping the microenvironment, or in infiltrating immune cells, which then establish immune tolerance? Clarifying this sequence is essential for determining optimal therapeutic timing and targets. Similarly, the role of stereoisomers—L-lactate versus D-lactate—remains poorly understood. While L-lactate predominates in mammalian metabolism, microbial-derived D-lactate may also contribute to host protein lactylation. Whether these isomers yield distinct functional outcomes is an open question with implications for microbiome–cancer interactions.

Finally, methodological challenges hinder progress. Detection of lactylation remains technically demanding, with antibody-based assays lacking specificity and mass spectrometry limited by sensitivity. Standardized detection platforms are needed to translate lactylation from discovery biology into clinical biomarkers.

## Future directions

Future research must advance beyond descriptive studies toward mechanistic dissection and translational application. Comprehensive lactylome mapping across tumor types, disease stages, and treatment responses will be critical. Proteome-wide LC-MS/MS, integrated with chromatin immunoprecipitation and spatial transcriptomics, can reveal how lactylation distributes across histone and non-histone substrates, and how it shapes intratumoral heterogeneity. Such efforts will provide the blueprint for prioritizing lactylation events as therapeutic targets.

Equally urgent is the structural biology of lactylation. High-resolution cryo-EM and crystallography studies are needed to elucidate how lactylation alters protein conformation, stability, and protein–protein interactions. Without structural clarity, it remains difficult to predict which lactylation events drive oncogenesis and which are epiphenomena.

Moving beyond bulk tissue analysis, recent single-cell RNA sequencing (scRNA-seq) and spatial transcriptomics studies have begun to map the “lactylome” at cellular resolution, revealing that lactylation is not uniformly distributed but defines specific, therapy-resistant cell states (127).

First, cell-state specificity has been identified as a key driver of resistance. For instance, in bladder cancer, scRNA-seq analysis isolated a distinct subpopulation of cisplatin-resistant epithelial cells characterized by hyper-lactylation (specifically H3K18la) (128). This state was driven by the transcription factors YBX1 and YY1, which locked cells into a glycolytic and drug-resistant phenotype impossible to detect in bulk samples. Similarly, in acute myeloid leukemia (AML), single-cell profiling revealed that high “lactylation scores” were restricted to malignant progenitor populations, which correlated with the recruitment of Tregs and M2 macrophages, thereby creating a localized immunosuppressive niche (129).

Second, spatial transcriptomics has added a geographic dimension to these findings. In glioblastoma, multi-omics integration demonstrated that lactylation-high clusters (marked by S100A6 expression) are not randomly dispersed but are spatially confined to hypoxic tumor cores and perinecrotic regions (130). These “lactylation niches” spatially overlap with immunosuppressive macrophages, suggesting that metabolic crosstalk is strictly compartmentalized. In ovarian cancer, resistant subpopulations exhibiting high ALDH1A1 and S100A4 expression were found to co-localize with lactylation signals, linking metabolic reprogramming directly to spatial stemness niches (131, 132).

Therefore, future research must utilize these high-resolution technologies not just to catalogue modifications, but to dissect the spatial topography of lactylation. Identifying the specific “lactylation-high” subclones that survive therapy and seed metastasis will be critical for developing precision inhibitors that target the most dangerous tumor populations while sparing healthy tissue.

The microbiome represents a largely unexplored frontier. Commensal bacteria, particularly Lactobacillus species, produce D-lactate through fermentation. Whether microbial-derived lactate contributes to host protein lactylation remains unknown, but the possibility of microbiome–epigenome crosstalk is tantalizing. If validated, this could open new therapeutic avenues involving dietary modulation, probiotics, or microbiota-targeted drugs.

Clinical translation demands that lactylation be incorporated into biomarker development. Profiling lactylation in tumor biopsies and circulating tumor cells may yield predictive markers of immunotherapy response. Furthermore, embedding lactylation profiling in clinical trials of ICIs, CAR-T, or metabolic inhibitors will clarify whether lactylation can serve as a stratification tool. Ultimately, lactylation-based diagnostics could guide patient selection, identify resistance mechanisms, and inform combination regimens.

## Conclusion

Lactylation has emerged as a paradigm-shifting concept in cancer biology, embodying the convergence of metabolism, epigenetics, and immune regulation. By directly linking lactate accumulation to chromatin remodeling and protein regulation, lactylation explains how tumors exploit metabolic stress to drive adaptability, immune evasion, and therapy resistance.

Yet lactylation remains an unfinished story. Key questions regarding its enzymatic regulation, functional specificity, and therapeutic exploitability demand urgent answers. Its dualistic potential—sometimes oncogenic, sometimes immunostimulatory—remains underexplored. Methodological limitations continue to impede translation, and clinical validation has only begun.

Nevertheless, the trajectory of discovery is clear. Lactylation is not merely a biochemical curiosity but a metabolic language by which tumors orchestrate survival. Decoding and silencing this language may enable the next generation of therapies that integrate metabolic inhibition, epigenetic modulation, and immunotherapy. By recognizing lactylation as both a hallmark of tumor adaptation and a potential Achilles’ heel, the field now stands poised to convert a once-dismissed metabolite into a cornerstone of precision oncology.
